# Hypoxia regulates Th17/Treg balance by altering chromatin accessibility and gene expression

**DOI:** 10.1111/febs.70353

**Published:** 2025-12-23

**Authors:** Mariana Cázares‐Olivera, Shiyang Song, Sofia Ylinen, Talha Qureshi, Bin Li, Zhi Chen

**Affiliations:** ^1^ Faculty of Biochemistry and Molecular Medicine, Biocenter Oulu University of Oulu Finland; ^2^ Center for Immune‐Related Diseases at Shanghai Institute of Immunology, Department of Respiratory and Critical Care Medicine Shanghai Jiao Tong University School of Medicine China; ^3^ Department of Thoracic Surgery of Ruijin Hospital Shanghai Jiao Tong University School of Medicine China; ^4^ Department of Immunology and Microbiology Shanghai Jiao Tong University School of Medicine China

**Keywords:** chromatin accessibility, hypoxia, inflammation, Th17, Treg

## Abstract

Hypoxia plays an important role in multiple biological processes, including inflammation, and modulates the T helper 17 (Th17) and regulatory T‐cell (Treg) imbalance that often contributes to inflammatory diseases. Although the transcription factor (TF) hypoxia‐inducible factor 1‐alpha (HIF‐1α) drives gene expression changes that promote Th17 differentiation, whether hypoxia modulates chromatin openness in Th17 and Treg cells has not been well characterized. Here, we applied assay for transposase‐accessible chromatin using sequencing (ATAC‐seq) and RNA sequencing (RNA‐seq) to investigate how hypoxia alters the chromatin accessibility of Th17 and Treg cells, and how this correlates with the transcriptomic consequences that impact Th17/Treg balance. Our integrated analysis led to the identification of several factors that could contribute to shifting Treg differentiation toward a Th17 phenotype. Hypoxia induced extensive gene expression changes in Treg cells, compared with Th17, including a stronger upregulation of *Hif1a*, and increased expression of signal transducer and activator of transcription 3 (*Stat3*) mRNA and protein. This crosstalk between hypoxia and STAT3 in Treg cells suggests a previously unknown potential regulatory mechanism influencing Treg cell differentiation. Furthermore, we highlight TFs protein C‐ets‐1 (ETS1), interferon regulatory factor 1 (IRF1), runt‐related transcription factor 2 (RUNX2) and cyclic AMP‐dependent transcription factor ATF‐3 (ATF3), which could be relevant modulators of T helper (Th) cell differentiation in hypoxic environments, such as inflamed tissue. Our study shows that hypoxia contributes to regulating the Th17 and Treg balance by increasing accessibility of key loci that are involved in Th cell differentiation and favor a Th17 phenotype. Our results provide valuable insight into how hypoxia alters chromatin accessibility and gene expression of TFs that, in addition to HIF‐1α, could modulate Treg differentiation and shift the Th17/Treg balance in hypoxic environments.

## Graphical abstract

T‐cell differentiation into Th17 or iTreg cells is key to inflammation control. Integrated RNA‐seq and ATAC‐seq analyses reveal that hypoxia induces more extensive gene expression changes in iTreg cells. Elevated levels of Hif1a and Stat3, along with increased accessibility to key transcription factor motifs, may predispose these cells toward alternative differentiation pathways.

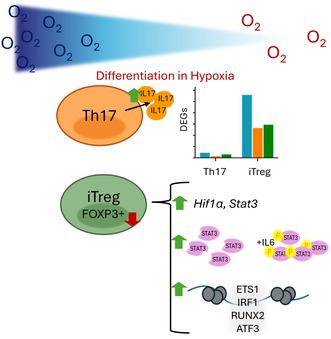

**Abbreviations**
AHR: aryl hydrocarbon receptor
AKT: Protein kinase B
AKT3: AKT serine/threonine kinase 3
ARNT: aryl hydrocarbon receptor nuclear translocator
ATAC‐seq: assay for transposase‐accessible chromatin using sequencing
ATF3: cyclic AMP‐dependent transcription factor ATF‐3
BCL2: B‐cell lymphoma 2 gene
BORIS: brother of regulator of imprinted sites
CAMK2G: calcium/calmodulin‐dependent protein kinase II gamma
CDKN1B: cyclin‐dependent kinase inhibitor 1b
CREBBP: CREB‐binding protein
CTCF: 11‐zinc finger protein or CCCTC‐binding factor
CUGBP2: CUG‐binding protein 2
CUL2: cullin 2
CXCR4: CXC chemokine receptor type 4
CYP1A1: cytochrome p450 family 1 subfamily a member 1
DEG: differential gene expression
ETS1: protein c‐ets‐1
ETV6: ETS variant transcription factor 6
EZH2: enhancer of zeste homolog 2
FOXO: forkhead box protein O
FOXO6: forkhead box O6
FOXP3: forkhead box P3
GLUT1: glucose transporter type 1
glycoPER: glycolytic proton efflux rate
H: hypoxia
HIF: hypoxia‐inducible factor
HIF‐1α: hypoxia‐inducible factor 1, alpha subunit
HIF‐1αi: HIF‐1α Inhibitor
IFNGR1: interferon gamma receptor 1
IFNGR2: interferon gamma receptor 2
IGF1R: insulin‐like growth factor I receptor
IL‐17: interleukin‐17
IL‐2: interleukin‐2
IL‐6: interleukin‐6
INSR: insulin receptor
IRF1: interferon regulatory factor 1
iTreg: induced T regulatory
iTreg: induced T regulatory
JAK2: janus kinase 2
MIAT: myocardial infarction associated transcript
mTOR: mechanistic target of rapamycin kinase
N: normoxia
OCR: oxygen consumption rate
OXPHOS: oxidative phosphorylation
PCA: principal component analysis
PER: proton efflux rate
PI3K: phosphoinositide 3‐kinase
PLCG1: phospholipase C, gamma 1
PREX1: phosphatidylinositol‐3,4,5‐trisphosphate‐dependent Rac exchange factor 1
RARA: Retinoic acid receptor alpha
RICTOR: RPTOR independent companion of mtor
RNA‐seq: RNA sequencing
RORA: RAR‐related orphan receptor alpha
RORγt: RAR‐related orphan receptor gamma t
RUNX2: runt‐related transcription factor 2
SMURF1: SMAD specific E3 ubiquitin protein ligase 1
STAT: signal transducer and activator of transcription
STAT3: signal transducer and activator of transcription 3
STAT3i: STAT3 inhibitor
STAT5: signal transducer and activator of transcription 5
STRING: search tool for the retrieval of interacting genes/proteins
TCR: T‐cell receptor
TF: transcription factor
Th: T helper
Th1: T helper 1
Th17: T helper 17
Th2: T helper 2
TNFRSF8: tumor necrosis factor receptor superfamily, member 8
TNFSF4: tumor necrosis factor (ligand) superfamily, member 4
Treg: T regulatory
TSS: transcription starting sites
VDR: vitamin D receptor
VEGF: vascular endothelial growth factor

## Introduction

Imbalance between suppressive T regulatory (Treg) cells and pro‐inflammatory T helper 17 (Th17) cells has been implicated in the development of several inflammatory diseases, including multiple sclerosis, rheumatoid arthritis, and inflammatory bowel disease [1, 2, 3, 4]. A better understanding of the mechanisms regulating Th17 and Treg differentiation is needed to identify potential targets to modulate the Th17/Treg balance in inflammation. Decreased oxygen concentration is a characteristic of sites of immune activity [5] and is frequently observed in inflammatory disease [6, 7, 8]. Therefore, there is particular interest in elucidating the mechanisms underlying T helper (Th) cell differentiation in hypoxic microenvironments.

The process of Th cell differentiation can be regulated at transcriptional and epigenetic levels that permit the activation of lineage‐specific genes and silencing genes critical for alternate Th subsets. At the transcriptional level, the signal transducer and activator of transcription (STAT) family of transcription factors (TF) plays an essential role driving lineage‐specific gene expression programs during Th cell differentiation [9]. Epigenetic mechanisms such as the post‐translational modification of histones, regulate the accessibility of key Th subset‐specific cytokine loci, facilitating or obstructing binding of TFs for gene transcription [10, 11]. Extensive epigenetic modifications and chromatin remodeling take place during Th cell activation [12], and differentiation [13], and are involved in Th cell function, stability, and plasticity [14]. Chromatin remodeling during Th cell differentiation is affected by TFs, such as STAT3 [15, 16] and STAT5 [17], which are required for Th17 and Treg cell differentiation, respectively. Interestingly, differences in the chromatin accessibility profiles of Th cell subsets may influence the ease with which Th phenotypes are altered by extracellular stimuli. For instance, differing chromatin accessibility landscapes in naïve CD4+ T cells contribute to the propensity of acquiring either an effector Th or a follicular helper T‐cell phenotype during early T‐cell fate determination in response to T‐cell receptor (TCR) stimulation [18].

Additional factors within the inflammatory microenvironment, such as hypoxia, impact the Th17/Treg balance. Previous studies have shown that the limited oxygen availability in inflamed tissue leads to stabilization of HIF‐1α, the oxygen‐sensitive subunit of the hypoxia‐inducible factor (HIF) TF, and induces HIF signaling, promoting Th17 differentiation at the expense of Treg cell differentiation [19, 20, 21]. Furthermore, an underlying connection is observed between STATs and HIF signaling, as STAT3 drives the upregulation of HIF‐1α to support Th17 differentiation [20].

In cancer cells, hypoxia not only induces transcriptional changes, but also causes changes in chromatin accessibility [22]. Current studies on chromatin remodeling in T cells have focused on how hypoxia within the tumor microenvironment affects T‐cell exhaustion [23, 24], and CD8+ T‐cell effector function in cancer [25]. Additionally, it has been reported that the HIF‐1α‐dependent glycolysis pathway impacts epigenetic remodeling during Tfh differentiation [26]. However, how hypoxia alters the chromatin accessibility landscape and contributes to regulating the Th17/Treg balance is still not fully understood.

Targeting hypoxia signaling has been a topic of interest in research as it provides an attractive possibility to treat a variety of hypoxia‐associated pathologies, including cancer, cardiovascular, and metabolic disease [27]. Roxadustat, a HIF‐prolyl‐hydroxylase inhibitor that prevents the degradation of HIF‐1α, has been approved in China for the treatment of chronic kidney disease to increase erythropoiesis in patients with anemia [28]. Although the field is promising, it is critical to understand how the hypoxia response contributes to disease progression in different contexts, whether upregulation or suppression is needed in each case, and explore whether there are more specific targets within the hypoxia response network that could be modulated. Therefore, further characterization of the mechanisms involved in hypoxia is critical. In the case of Th cells and inflammatory disease, there is a need to better understand how hypoxic environments might prime cell differentiation toward distinct T effector phenotypes, and gauge potential unintended consequences in the immune system when manipulating the hypoxia response.

Here, we investigate how hypoxia regulates Th17/Treg balance by altering chromatin accessibility and gene expression. Using assay for transposase‐accessible chromatin using sequencing (ATAC‐seq), we mapped genome‐wide open chromatin regions during Th17 and Treg differentiation under low‐oxygen culture conditions. Our study provides valuable data to help identify accessible loci that are available for transcriptional regulation and could contribute to modulating Th cell responses in hypoxia, a biologically relevant context for inflammatory disease. Integrated ATAC‐seq and RNA‐seq analyses allowed the identification of potential TFs that contribute to modulating the Th17/Treg balance under hypoxia toward a Th17 phenotype. Of interest, we found that hypoxia induces changes in chromatin accessibility at the *Stat3* loci and upregulates STAT3 expression at the RNA and protein level in Treg cells, which may contribute to modulating differentiation. Our study provides insight into how hypoxia affects Th17/Treg balance.

## Results

### ATAC‐seq analysis of Th17/iTreg subset‐specific gene loci shows open chromatin regions available for transcriptional regulation under hypoxia

**Fig. 1:**
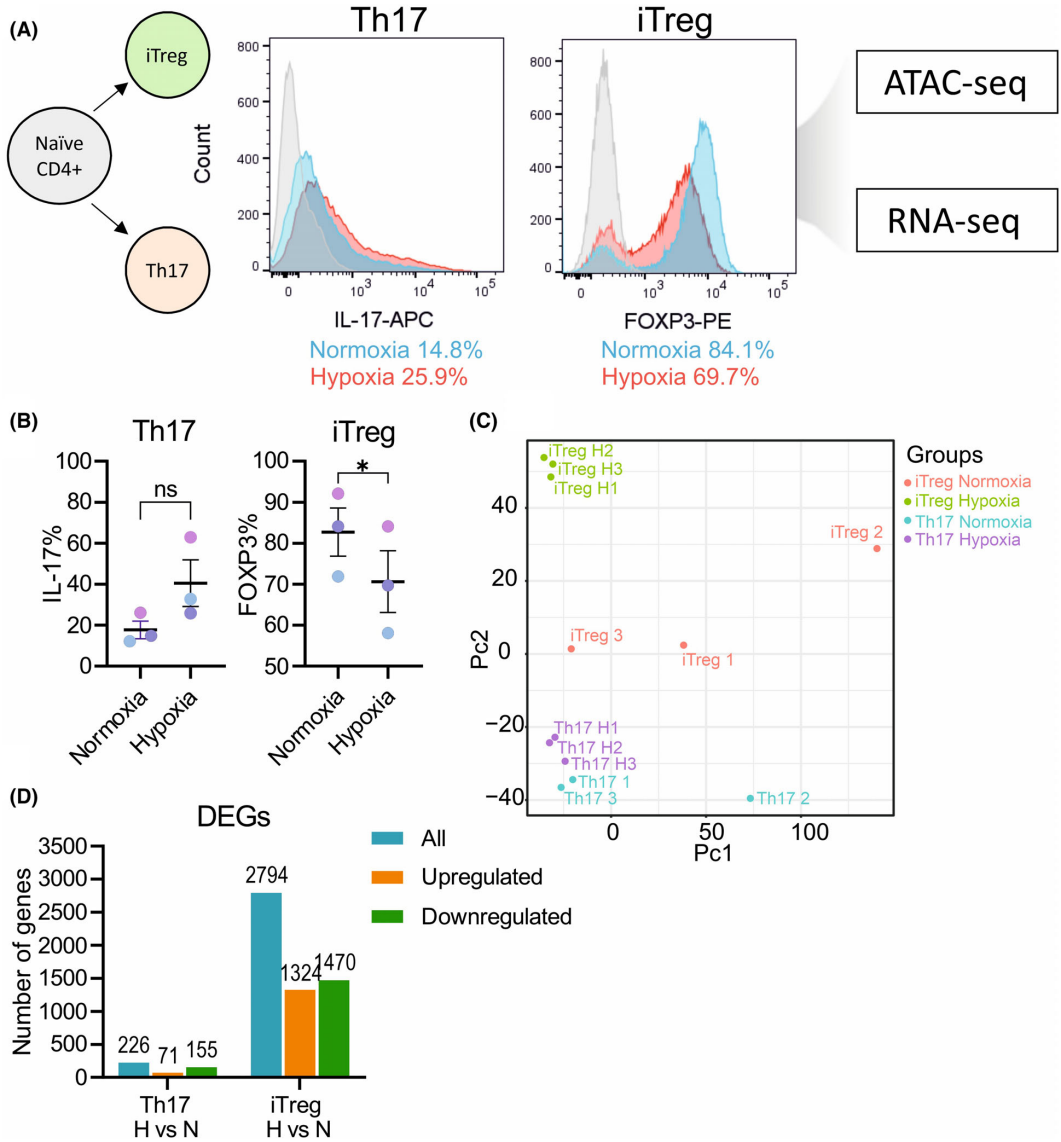
Experimental setup and representative intracellular staining of differentiated mouse T helper 17 (Th17) and induced Treg (iTreg) cells for RNA‐seq and assay for transposase‐accessible chromatin using sequencing (ATAC‐seq) analysis. (A) Diagram of the experimental setup of T helper (Th) subset differentiation for ATAC‐seq and RNA‐seq analysis. Naïve CD4+ cells isolated from mouse spleen and lymph nodes were cultured for 3 days in normoxia and hypoxia under Th17 and iTreg differentiation conditions. Th17 and iTreg cell differentiation was assessed by intracellular staining of IL‐17 and intranuclear staining of forkhead box P3 (FOXP3), respectively, and measured by flow cytometry. Cells were restimulated with Phorbol 12‐myristate 13‐acetate (PMA), Ionomycin and Brefeldin A for intracellular cytokine staining. A representative histogram from intracellular staining of Th17 (IL‐17+) and intranuclear staining of iTreg (FOXP3+) cells is shown, overlaying unstained (gray), normoxia (blue) and hypoxia (red) condition. Samples from independent Th17 and iTreg differentiation cultures were used for ATAC‐seq (*n* = 3) and RNA‐seq (*n* = 3) analysis. (B) Quantification of FOXP3+ cells in iTreg and IL‐17+ cells in Th17 condition measured by flow cytometry from three independent experiments. The mean is marked by a black horizontal line, and error bars indicate s.e.m. The statistical analysis was performed using paired t‐test where *P ≤ 0.05. (C) Principal component analysis of the RNA‐seq samples (*n* = 3). Th17 and iTreg cells differentiated in normoxia (N) and hypoxia (H) from three independent experiments are numbered 1 to 3. (D) Bar graph showing the number of differentially expressed genes (DEGs) identified from the RNA‐seq analysis of Th17 and iTreg cells (*n* = 3) in hypoxia compared with normoxia (H vs. N), including the total number of DEGs (blue), upregulated genes (orange), and downregulated genes (green). Treg, T regulatory.

To characterize whether reduced oxygen level directly impacts epigenetic remodeling cascades in Th17 and induced Treg (iTreg) cells, we utilized ATAC‐seq and RNA‐seq technologies to map accessible chromatin regions and gene expression changes in *in vitro* polarized Th17 and iTreg cells under normoxia and hypoxia conditions (Fig. 1A). Firstly, hypoxia increased the proportion of interleukin‐17 (IL‐17)‐producing cells under Th17‐polarizing condition, whereas the percentage of forkhead box P3 (FOXP3)‐positive cells decreased, indicating that hypoxia shifts the Th cell differentiation balance in favor of a Th17 phenotype while suppressing iTreg differentiation in mouse CD4+ cells (Fig. 1B).

**Fig. 2:**
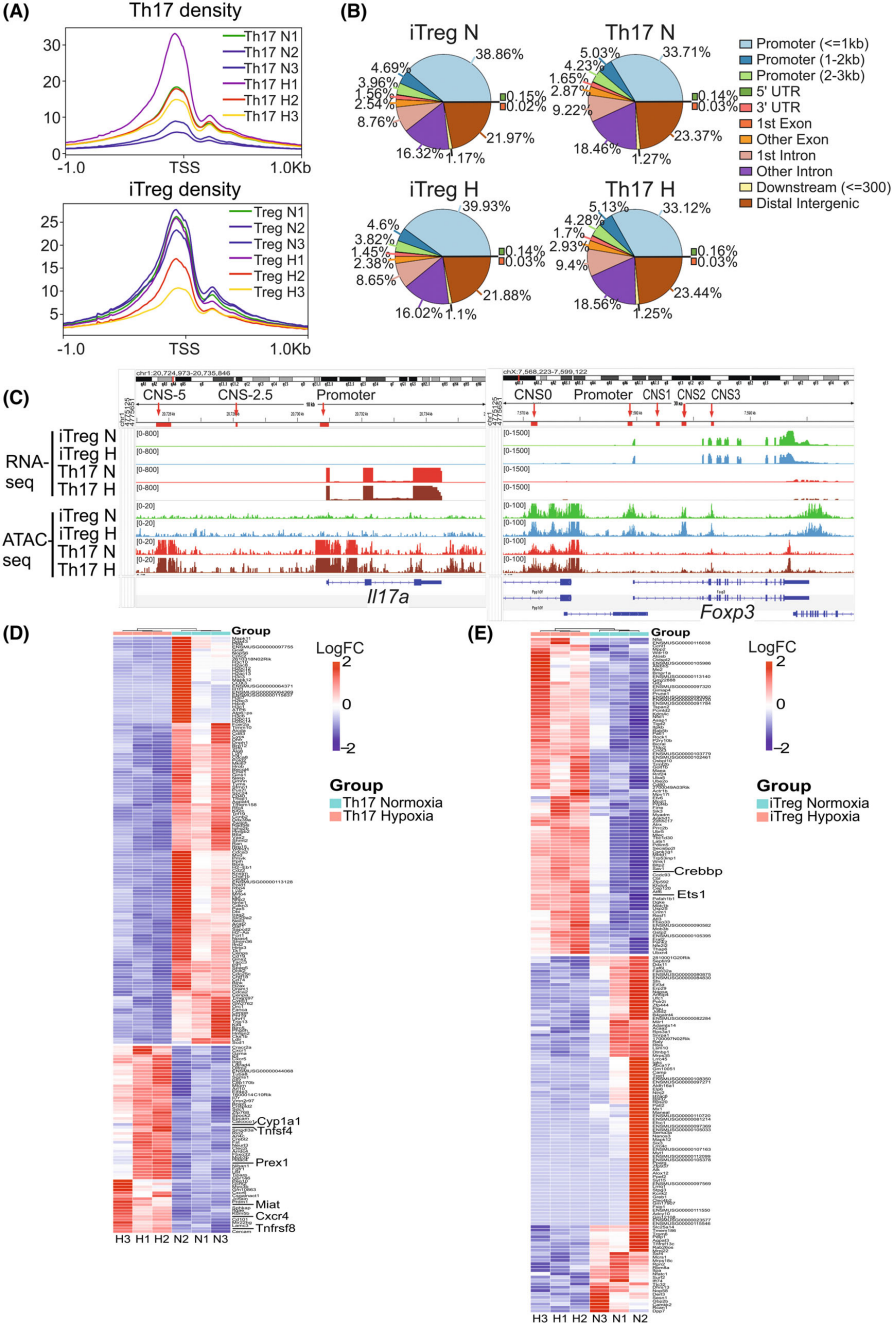
Assay for transposase‐accessible chromatin using sequencing (ATAC‐seq) and RNA‐seq analysis provides insight into the regulation of key loci for T helper 17 (Th17) and Treg, T regulatory (Treg) differentiation in hypoxia. (A) Histograms of peak‐transcription starting sites (TSS) distance distribution of ATAC‐seq analysis. The signal distribution around ± 1 kb from the TSS (transcription start site) is shown for three replicates (1–3) of Th17 and induced Treg (iTreg) cells differentiated in normoxia (N) and hypoxia (H). The horizontal axis represents position relative to the gene TSS and the vertical axis represents peak density. TSS of every peak‐related gene is detected by PeakAnnotator [84]. (B) Pie chart of the peak distribution where peaks are annotated to functional regions across samples. The correspondence between the peak and each functional area is in the order of priority of promoter, 5′UTR, 3′UTR, Exon, Intron, Downstream, and Intergenic. (C) mRNA expression and chromatin accessibility of *Il17a* and *Foxp3*. The ATAC‐seq and RNA‐seq Integrative Genomics Viewer tracks at the *Il17a* and *Foxp3* loci are shown from Th17 and iTreg cells differentiated under normoxia (N) and hypoxia (H). For the *Foxp3* locus, CNS0, CNS1, CNS2, and the promoter loci were marked [85]. For the *Il17a* locus, CNS located 5‐kb upstream, CNS1 at 2.5‐kb upstream, and the promoter loci were marked. (D) Heatmap showing the top 200 differentially expressed genes in Th17 cells and (E) iTreg cells under hypoxia and normoxia conditions. *Foxp3*, forkhead box P3.

To identify changes in chromatin accessibility caused by hypoxia in Th17 and iTreg conditions, we performed ATAC‐seq combined with RNA‐seq analysis (Fig. 1). Principal component analysis (PCA) from our RNA‐seq data revealed that while Th17 cells differentiated in normoxia and hypoxia clustered together with a similar gene expression profile, iTreg cells under hypoxia conditions differed from all other groups (Fig. 1C). Consistently, analysis of differentially expressed genes (DEGs) further revealed that hypoxia induces more extensive transcriptional changes in iTreg cells than in Th17 cells (Fig. 1D and Tables S1, S2). To further investigate how hypoxia modulates gene expression, we examined the chromatin landscape using our ATAC‐seq analysis. We first examined the genomic distribution of open chromatin reads and observed that most of the identified peaks were located around transcription starting sites (TSS), consistent with open regions that might be relevant for regulating gene transcription (Fig. 2A). We mapped the peak distribution in each Th cell differentiation condition in normoxia and hypoxia and observed as expected that the highest proportion of peaks was located within 1‐kb promoter regions, with similar peak distribution between normoxia and hypoxia conditions (Fig. 2B). Notably, we also observed that 16–19% of the peaks were mapped in other introns and 21–24% of the peaks were in distal intergenic regions, which may represent enhancers.

RNA‐seq data showed that the expression of *Il17a* was detected in the Th17 cells but not in the iTreg cells and *Foxp3* expression was detected in iTreg cells but not in Th17 cells, confirming the cells were polarized to the expected phenotype. Congruently, open chromatin regions at the *Il17a* locus were detected in Th17 cells. Interestingly, we observed that iTreg cells had open chromatin regions both at the *Il17a*, and the *Foxp3* loci, suggesting a permissive region that can be accessed to regulate iTreg cell fate (Fig. 2C). However, hypoxia conditions did not alter either the chromatin accessibility at the *Foxp3* and the *Il17a* loci, or their gene expression at the RNA level. Our findings are in line with previous findings showing that FOXP3 regulation under hypoxia conditions primarily occurs at the protein level [20]. Interestingly, the accessibility of the *Foxp3* locus remains unchanged under both normoxia and hypoxia conditions, suggesting that it is readily available for transcriptional regulation regardless of oxygen conditions. Differential gene expression analysis revealed that in Th17 cells, hypoxia predominantly led to gene suppression rather than induction (Figs 1D and 2D). In contrast, iTreg cells exhibited a more balanced response, with upregulated and downregulated genes being equally represented (Figs 1D and 2E). This suggests that iTreg cells undergo broader transcriptional changes in response to hypoxia.

### Chromatin accessibility profile changes in Th17 cells under hypoxia condition

**Fig. 3:**
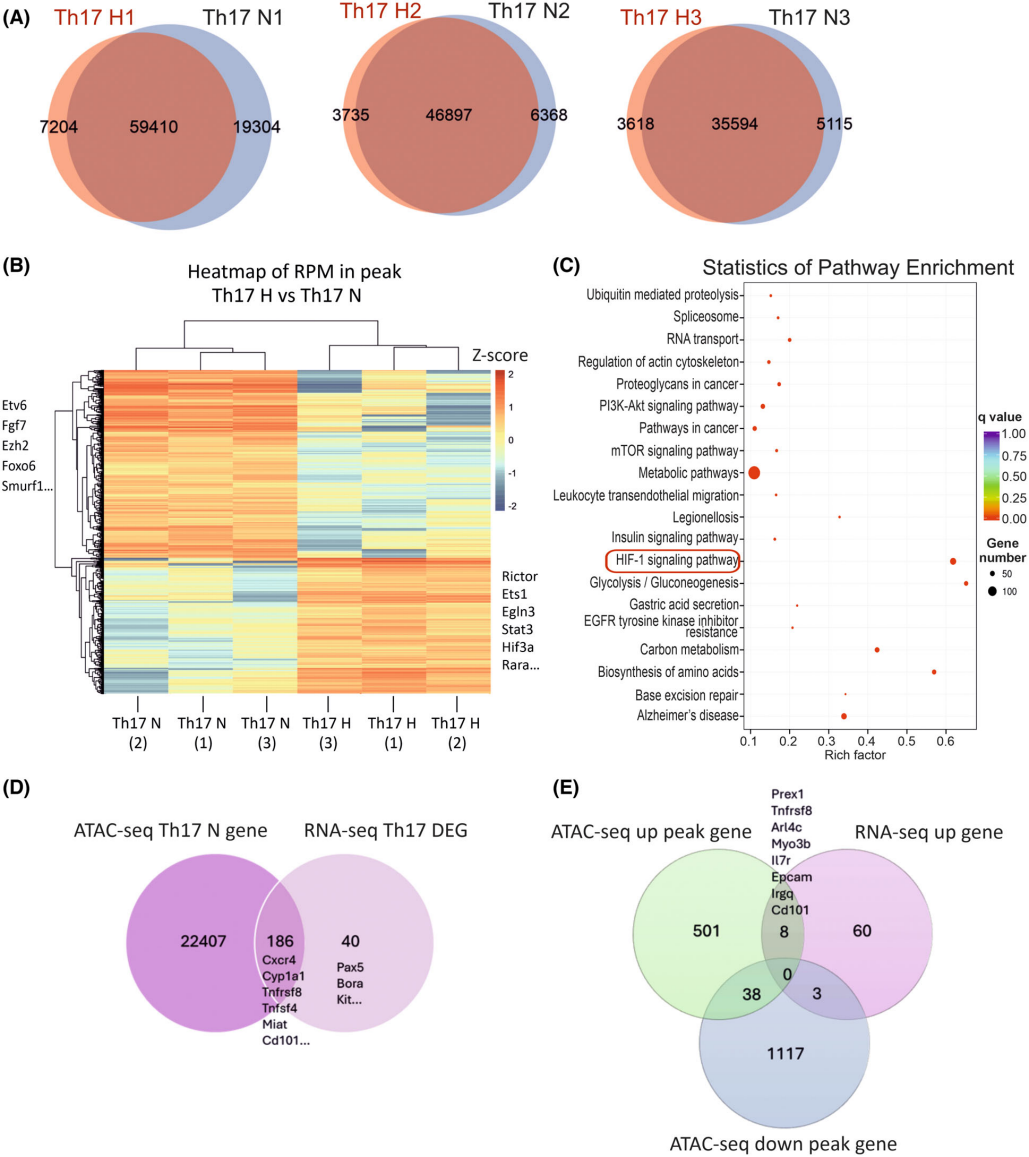
Hypoxia causes chromatin accessibility changes in T helper 17 (Th17) cells. (A) Chromatin accessibility peak overlaps in normoxia and hypoxia of Th17 samples. Venn diagrams showing the differential assay for transposase‐accessible chromatin using sequencing (ATAC‐seq) peaks detected in hypoxia (H) and normoxia (N) from three replicates (1–3). (B) Hierarchical clustering from ATAC‐seq peak enrichment analysis of Th17 hypoxia (H) and normoxia (N) groups (*n* = 3). Hierarchical clustering is performed based on the RPM value (the number of reads enriched in peak area per million reads) in different samples. Genes of interest associated with the enriched chromatin peaks are listed next to each experimental group. (C) ATAC‐seq pathway enrichment analysis from differentially accessible regions in Th17 hypoxia condition, compared with Th17 normoxia. KEGG enrichment analysis (including increased and decreased accessible peaks) for the differential peak‐related genes is shown, where adjusted *P*‐value is indicated by color and the number of enriched genes within the pathway are indicated by dot size. A red box highlights the enrichment of the hypoxia‐inducible factor 1 (HIF‐1) signaling pathway by hypoxia. (D) Correlation between Th17 accessible chromatin regions and differentially expressed genes (DEGs). The Venn diagram shows the overlap between RNA‐seq DEG induced by hypoxia and accessible peaks within genes in Th17 cells from normoxia ATAC‐seq analysis. Selected genes with differential gene expression from RNA‐seq analysis overlapping with accessible peaks in normoxia are marked at the diagram overlap. (E) Analysis of ATAC‐seq peaks overlapping with RNA‐seq DEGs in hypoxia Th17 samples. Venn diagram showing the ATAC‐seq peaks of differentially accessible chromatin regions within genes with RNA‐seq differentially upregulated genes (up gene) in Th17 hypoxia samples. The peaks within genes detected from ATAC‐seq analysis include increased (up peak) and decreased (down peak) accessible peaks in response to hypoxia in Th17 samples. Genes with increased accessibility from ATAC‐seq analysis (up peak) and with increased gene expression from RNA‐seq analysis (up gene) are marked at the diagram overlap.

To better understand the impact of hypoxia on Th17 cell differentiation, we performed further analysis of our ATAC‐seq data to profile chromatin accessibility under hypoxia and identify associated gene changes in Th17 cells (Table S3). We observed that a majority of the detected peaks in the hypoxia and normoxia conditions were overlapping (Fig. 3A), corresponding to regions that remain accessible in both oxygen culture conditions. The effect of hypoxia on chromatin accessibility in Th17 was seen from clustering analysis of accessible chromatin regions in Th17 cells under hypoxia and normoxia (Fig. 3B). Hypoxia reduced the number of accessible regions in Th17 cells, including *Etv6*, *Ezh2*, *Foxo6* and *Smurf1* (Fig. 3B). Notably, as an epigenetic regulator, enhancer of zeste homolog 2 (Ezh2) was previously reported to inhibit the expression of Th1, Th2, and Th17 lineage signature cytokines, including IL‐17 and promoting the expression of FOXP3 and the activation of Treg cells [29, 30]. A recent study reported that SMAD‐specific E3 ubiquitin protein ligase 1 (Smurf1) also inhibits Th17 differentiation and increases the Treg/Th17 imbalance [31]. Importantly, several open chromatin peaks associated with important genes for Th17 cell differentiation were found increased, such as *Rara*, *Stat3*, and *Rictor*. These genes code for proteins implicated in the regulation of Th17 differentiation. STAT3 is an essential TF in Th17 differentiation [17, 32]. RPTOR independent companion of MTOR (RICTOR) is a scaffolding protein of the mechanistic target of rapamycin (mTOR) complex 2. While the mTOR complex functions as an environmental sensor and metabolic regulator that promotes glycolysis thus influencing Th cell differentiation, the mTOR complex 2 specific effects on Th cell differentiation require further characterization [33]. The retinoic acid receptor alpha (RARA), which mediates induction of iTreg cells and suppression of Th17 via retinoic acid signaling [34, 35, 36], and its ligand‐independent activity has been linked to T‐cell metabolic adaptations during activation [37]. Hypoxia increased accessibility to these loci allowing potential regulation of Th17 cell differentiation. Pathway enrichment analysis of differentially accessible chromatin regions under hypoxia and normoxia conditions in Th17 cells confirmed the upregulation of HIF‐1 signaling, which consisted of genes with increased accessibility (Fig. 3C and Table S4), together with the enrichment of genes important for metabolism, glycolysis, biosynthesis of amino acids, phosphoinositide 3‐kinase/protein kinase B (PI3K‐Akt) and mTOR signaling pathways. Th17 cells use glycolysis as a main metabolic pathway that supports cell differentiation and function [21]. These results suggest that gene accessibility changes contribute to enabling the metabolic and transcriptional shift required to support a Th17 phenotype. Next, we examined the correlation between open chromatin regions and DEGs in Th17 cells and found that most of the accessible regions did not correspond with gene expression changes (Fig. 3D). On the contrary, as expected, the majority of the DEGs, including *Cyp1a1*, *Cxcr4*, *Tnfsf4*, and *Miat*, were associated with regions of open chromatin (Fig. 3D). Further analysis of the relationship between differentially accessible regions and upregulated genes in Th17 cells under hypoxia conditions revealed that only eight upregulated genes were associated with increased chromatin accessibility, including *Prex1* and *Tnfrsf8* (Fig. 3E). This suggests that even though many chromatin regions are accessible, hypoxia only makes a modest contribution in inducing transcription changes in Th17 differentiation. These accessible regions may be ‘primed’ but require specific signals other than hypoxia to become transcriptionally active.

### Hypoxia causes chromatin accessibility changes in iTreg cells

**Fig. 4:**
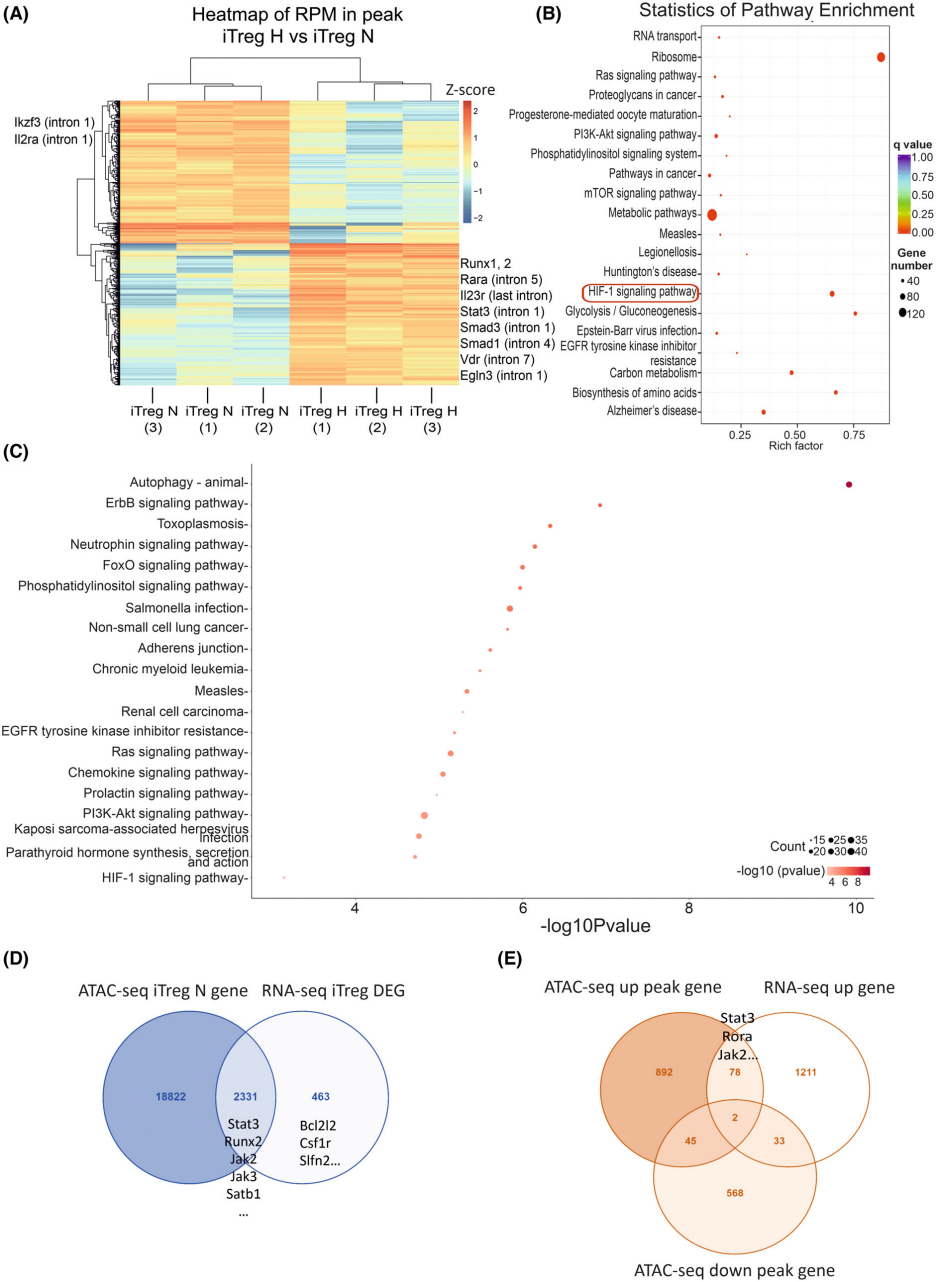
Correlation between chromatin openness of induced Treg (iTreg) cells and gene expression changes to hypoxia. (A) Hierarchical clustering from assay for transposase‐accessible chromatin using sequencing (ATAC‐seq) peak enrichment analysis of iTreg hypoxia (H) and normoxia (N) groups (*n* = 3). Hierarchical clustering is performed based on the RPM value (the number of reads enriched in peak area per million reads) in different samples. Genes of interest associated with the enriched chromatin peaks are listed next to each experimental group. (B) ATAC‐seq pathway enrichment analysis from differentially accessible regions in iTreg hypoxia condition, compared with iTreg normoxia. KEGG enrichment analysis (including increased and decreased accessible peaks) for the differential peak‐related genes is shown, where adjusted p‐value is indicated by color and the number of enriched genes within the pathway are indicated by dot size. A red box highlights the enrichment of the hypoxia‐inducible factor 1 (HIF‐1) signaling pathway by hypoxia. (C) RNA‐seq pathway enrichment analysis from upregulated differentially expressed genes (DEGs) in iTreg hypoxia compared with iTreg normoxia conditions. KEGG enrichment analysis for the differential peak‐related genes is shown, where log adjusted *P*‐value is indicated by color and the number of enriched genes within the pathway are indicated by dot size. (D) Correlation between iTreg accessible chromatin regions and DEGs. The Venn diagram shows the overlap between RNA‐seq DEG induced by hypoxia and accessible peaks within genes in iTreg cells from normoxia ATAC‐seq analysis. Selected DEGs from RNA‐seq analysis overlapping with accessible peaks in normoxia are marked at the diagram overlap. (E) Analysis of ATAC‐seq peaks overlapping with RNA‐seq DEGs in hypoxia iTreg samples. Venn diagram showing the ATAC‐seq peaks of differentially accessible chromatin regions within genes with RNA‐seq differentially upregulated genes (up gene) in iTreg hypoxia samples. The peaks within genes detected from ATAC‐seq analysis include increased (up peak) and decreased (down peak) accessible peaks in response to hypoxia in iTreg samples. Selected genes with increased accessibility from ATAC‐seq analysis (up peak) and with increased gene expression from RNA‐seq analysis (up gene) are marked at the diagram overlap. Treg, T regulatory.

Since we detected a consistent reduction of FOXP3 protein in iTreg cells cultured under hypoxia compared with normoxia conditions (Fig. 1B), we investigated the impact of hypoxia on chromatin accessibility and transcriptome in iTreg cells. Clustering analysis between iTreg cells in hypoxia and normoxia conditions showed a similar distribution of increased and reduced peaks in these two conditions (Fig. 4A). Pathway enrichment analysis confirmed the upregulation of the HIF‐1 signaling and enrichment in metabolic pathways, glycolysis, biosynthesis of amino acids, and the PI3K‐Akt/mTOR signaling pathways (Fig. 4B), which were also enriched in Th17 cells. Metabolism has an important role in Th cell differentiation, and the enriched pathways could be relevant for the regulation of the Th17/Treg balance by hypoxia. Genes associated with the enriched HIF‐1 signaling pathway included *Crebbp*, *Cdkn1b*, *Ifngr1*, *Ifngr2*, *Insr*, *Cul2*, *Stat3*, *Arnt*, *Hif1a*, *Igf1r*, *Vegfa*, *Akt3*, *Bcl2*, *Plcg1*, and *Camk2g* (Table S5). In addition, we identified the upregulation of pathways involved in cancer, forkhead box protein O (FoxO) signaling, and Th1/Th2 cell differentiation. Hypoxia caused more upregulated pathways compared with the downregulated pathways (Tables S5 and S6). Of interest, we observed downregulated oxidative phosphorylation (OXPHOS). OXPHOS is known to support the metabolic demands of Treg cells, particularly for maintaining their suppressive function and lineage stability. A decrease in OXPHOS may suggest impaired mitochondrial fitness, which could compromise iTreg functionality or promote plasticity toward pro‐inflammatory phenotypes. Importantly, pathway enrichment analysis of upregulated genes from the RNA‐seq data also confirmed significant enrichment in the HIF‐1 signaling pathway, as well as PI3K‐Akt, and phosphatidylinositol signaling pathways (Fig. 4C).

While the effects of hypoxia and HIF‐1α on Th17 differentiation have been extensively studied [20, 21, 38, 39], the mechanisms regulating iTreg differentiation in hypoxia are less clearly understood. To understand how open chromatin regions correlate with changes in gene expression under hypoxia, we integrated our RNA‐seq and ATAC‐seq datasets. Indeed, 83.4% of the DEGs caused by hypoxia in iTreg cells overlapped with the open chromatin regions of the iTreg cells (Fig. 4D and Table S7), indicating the loci of these genes are open for possible modification under hypoxia conditions which resulted in gene expression changes. Next, we analyzed the differentially accessible ATAC‐seq peaks whose intensity was increased or decreased in response to hypoxia and compared them with the upregulated and downregulated genes in iTreg cells detected by RNA‐seq. Among the increased open chromatin peaks annotated genes in iTreg cells, we found 80 of them were overlapping with increased gene expression under hypoxia (Fig. 4E). Notably, these include multiple genes involved in Th cell fate, such as *Rora, Stat3*, and *Jak2* (Fig. 4E), suggesting that increased accessibility at these loci could contribute to regulating iTreg cell differentiation.

### Stronger effect of hypoxia in regulating iTreg cell differentiation

**Fig. 5:**
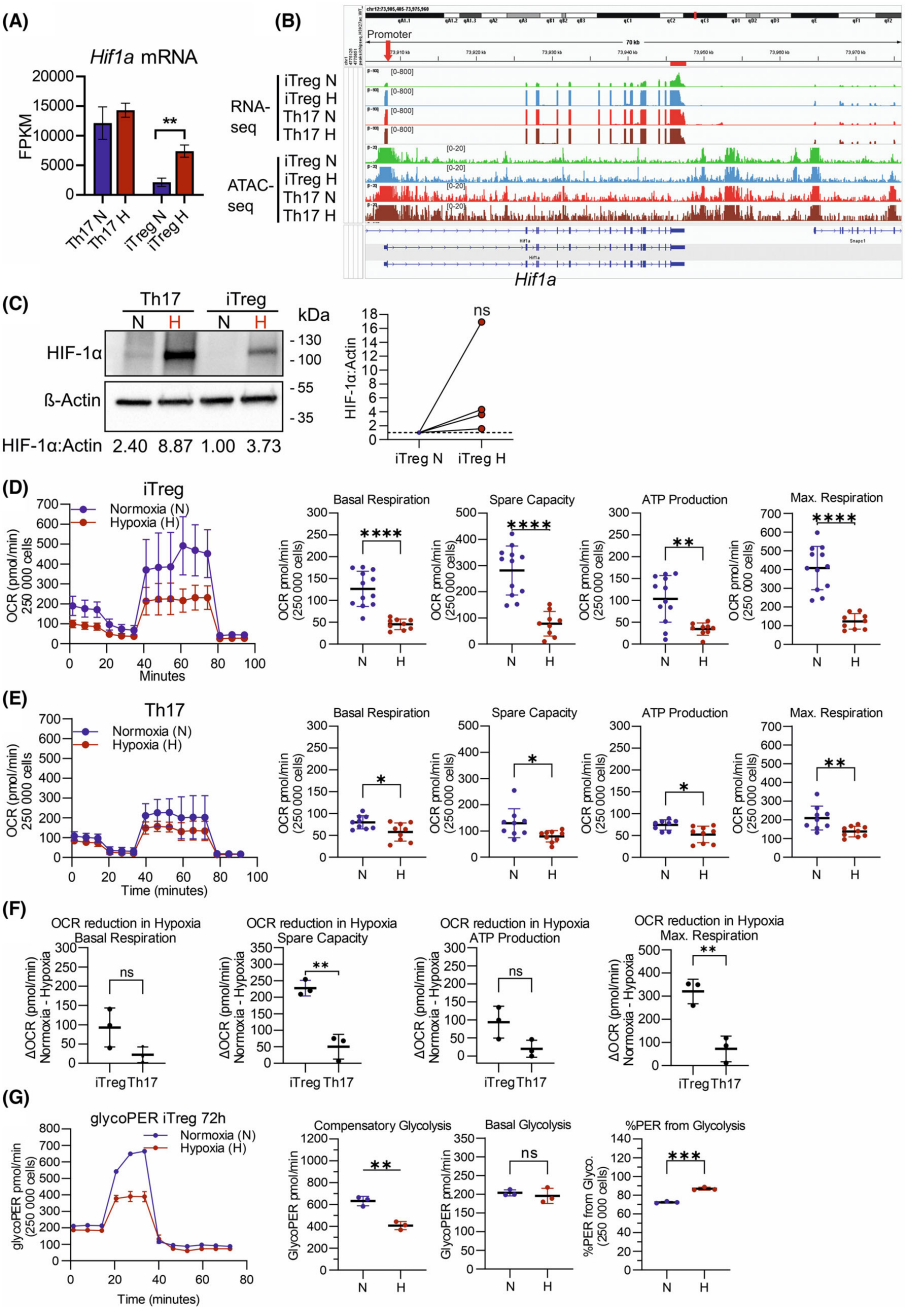
Stronger effect of hypoxia in induced Treg (iTreg) *Hif1a* gene expression, and suppression of mitochondrial metabolism. (A) Bar graph of *HIF1a* fragments per kilobase million (FPKM) values from RNA‐seq analysis of T helper 17 (Th17) and iTreg samples (*n* = 3). Differential expression analysis of two conditions/groups was performed using the DESeq2 R package, which provides statistical routines for determining differential expression in digital gene expression data using a model based on negative binomial distribution. Data presented as mean ± s.e.m where ** indicates FDR <0.01 comparing hypoxia (H) to normoxia (N). (B) mRNA expression and chromatin accessibility of *Hif1a* (*n* = 3). RNA‐seq genome tracks showing mRNA expression and assay for transposase‐accessible chromatin using sequencing (ATAC‐seq) genome track showing peaks in accessible regions and for Th17 and iTreg cells in normoxia (N) and hypoxia (H). (C) Western blot analysis of HIF‐1α protein in iTreg cells (*n* = 4). Representative western blot image (left) and relative protein quantification (right) from four independent experiments are shown for iTreg cells differentiated under normoxia (N) and hypoxia (H). β‐actin is used as a protein loading control and sample quantification is normalized to iTreg normoxia. The protein quantification of HIF‐1α relative to β‐actin (HIF‐1α : Actin) is indicated under each lane of the representative western blot. Statistical analysis was performed using one‐sample *t‐*test where ‘ns’ is *P* > 0.05. (D, E) Seahorse XF Cell Mito Stress Assay of Th17 (D, *n* = 3) and iTreg (E, *n* = 4) cells after 3 days differentiation under normoxia and hypoxia conditions. Three technical replicates are included in each Th17 and iTreg assay. The oxygen consumption rate (OCR) profile of iTreg (D) and Th17 (E) is shown as mean ± s.d. (left), and the corresponding quantified metabolic parameters basal respiration, spare respiratory capacity (spare capacity), ATP production, and maximal respiration (Max. Respiration) are shown to the right, including all technical replicates. The mean of the calculated parameters is marked by a black horizontal line, and error bars indicate s.d. Statistical analysis was performed for the calculated parameters using an unpaired t‐test, where *P ≤ 0.05, **P ≤ 0.01, ****P ≤ 0.0001. (F) Differences in OCR rates under hypoxic conditions compared with normoxia (∆OCR) for the iTreg and Th17 Seahorse XF Cell Mito Stress Assay quantified metabolic parameters (*n* = 3). ∆OCR values of basal respiration, spare respiratory capacity (spare capacity), ATP production, and maximal respiration (Max. Respiration), are shown, quantifying the shift in mitochondrial metabolism induced by hypoxia (normoxia OCR–hypoxia OCR). The mean is marked by a black horizontal line, and error bars indicate s.d. Statistical analysis was performed using unpaired *t*‐test where ‘ns’ is *P* > 0.05, and **P ≤ 0.01. (G) Seahorse XF Glycolytic Rate Assay of iTreg cells after 3 days differentiation under normoxia and hypoxic conditions. GlycoPER values are presented as mean ± s.d, from three technical replicates. Statistical analysis was performed using an unpaired *t*‐test, where ‘ns’ is p > 0.05, **P ≤ 0.01, ***P ≤ 0.001. HIF1, hypoxia‐inducible factor 1; Treg, T regulatory.

Although we observed that hypoxia affected both Th17 and iTreg cell differentiation (Fig. 1B), surprisingly, from transcriptomics data, we found that hypoxia caused significantly more gene expression changes in iTreg cells than in Th17 cells (Fig. 1D). HIF‐1α, a key TF that drives cellular adaptations to hypoxia, is also an important regulator of Th17 differentiation, and a direct STAT3 target gene [20, 21]. HIF‐1α has been previously reported to induce RAR‐related orphan receptor gamma *t* (RORγt) transcription in Th17 conditions and to target FOXP3 for proteasomal degradation [20]. We investigated whether there were differences in the regulation of HIF‐1α by hypoxia in Th17 and Treg cells. As expected, we observed from our RNA‐seq data that *Hif1a* was highly expressed in Th17 cells already under normoxia conditions. In iTreg cells, *Hif1a* expression was rather low in normoxia. However, hypoxia did not further enhance *Hif1a* mRNA levels in Th17 cells but significantly increased its expression in iTreg cells (Fig. 5A,B). Consistently, ATAC‐seq data showed that at normal oxygen conditions, the *Hif1a* locus is highly accessible in iTreg cells, which could facilitate the regulation of *Hif1a* expression (Fig. 5B). Hypoxia inhibits the degradation of HIF‐1α protein, and previous studies show that this mechanism contributes to Th17 differentiation [20]. We detected HIF‐1α protein expression by western blot. Indeed, we confirmed that a higher amount of HIF‐1α protein under hypoxia conditions compared with normoxia was observed in iTreg cells (Fig. 5C). However, this increase does not reach the expression levels observed in Th17 cells. This differential regulation is evident at both the transcript and protein levels, as shown in Fig. 5A,C. Taken together, we show that hypoxia decreases FOXP3 protein and causes more gene expression changes in iTreg than Th17 cells. These data suggest that hypoxia has a stronger effect in regulating iTreg differentiation, and the hypoxia‐induced HIF‐1α protein in iTreg cells may contribute to suppressing iTreg differentiation.

Given that metabolic pathways were enriched from our ATAC‐seq pathway analysis (Figs 3C and 4B), we examined how the metabolism of Th17 and Treg cells was affected after differentiation under hypoxia conditions. Mitochondrial function analysis of Th17 (Fig. 5D) and iTreg (Fig. 5E) cells using the Mito Stress assay revealed significantly impaired mitochondrial metabolism, including a significant reduction of basal respiration, spare respiratory capacity, ATP production, and maximal respiration in both subsets. Strikingly, the impact of hypoxia on mitochondrial metabolism was stronger in iTreg compared with Th17 cells, as evidenced by a greater reduction in the oxygen consumption rate (∆OCR) under hypoxia versus normoxia conditions. Hypoxia significantly reduced both the spare respiratory capacity and maximal respiration in iTreg, with a greater effect observed compared with Th17 cells (Fig. 5F). Given the stronger suppressive effect of hypoxia on mitochondrial metabolism in iTreg cells, we next performed a Glycolytic Rate Assay to further investigate their metabolic adaptation in iTreg cells after differentiation under normoxia and hypoxia conditions. We observed no significant differences in basal glycolysis, while compensatory glycolysis was reduced. Notably, hypoxia increased the contribution of glycolysis to the proton efflux rate (PER) in iTreg cells (Fig. 5G), indicating a metabolic shift toward glycolysis in iTreg cells under low‐oxygen conditions.

### Hypoxia induces expression of several key factors regulating Th17/Treg cell differentiation in iTreg cells

**Fig. 6:**
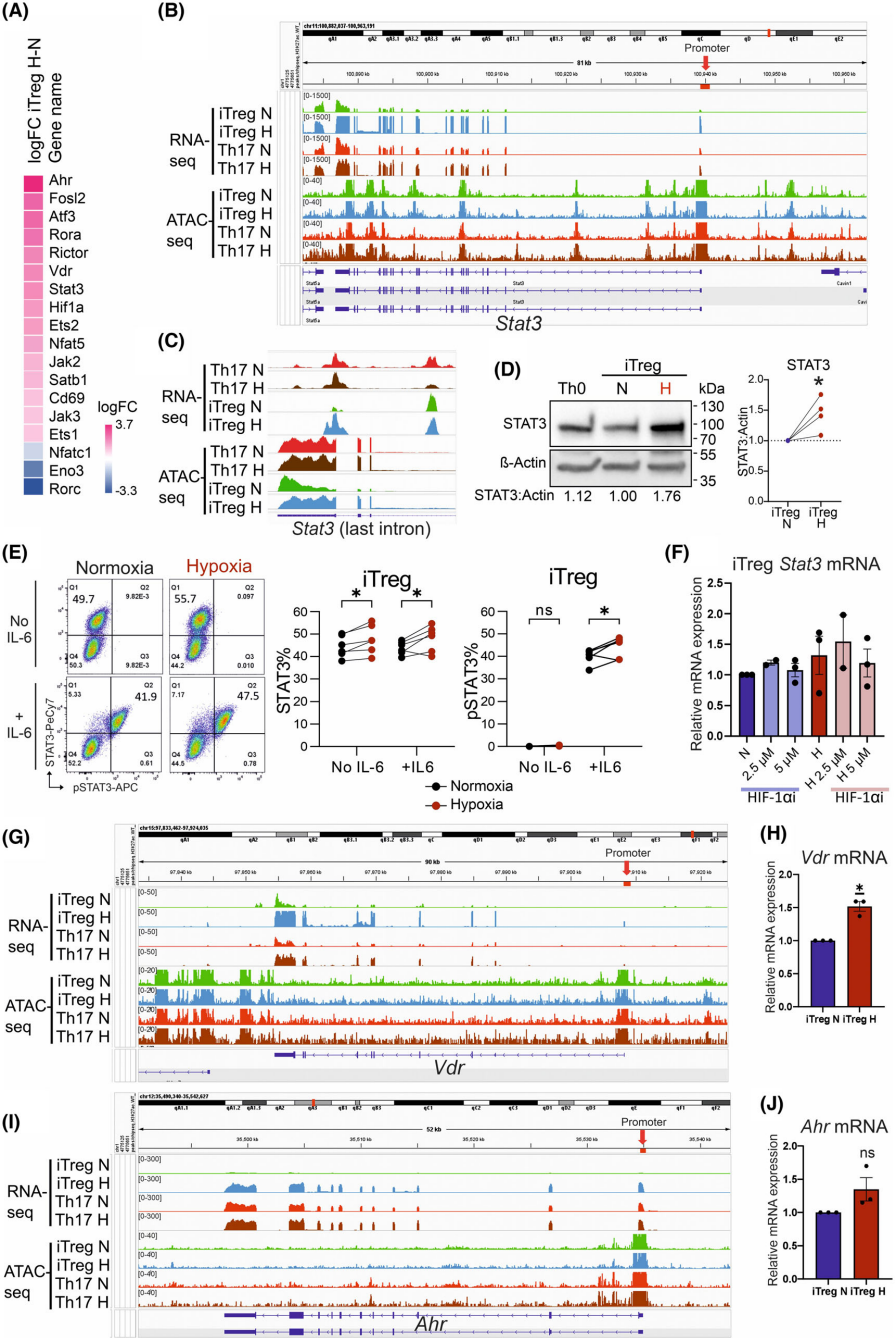
Hypoxia induces expression of *Stat3*, *Ahr*, and *Vdr* mRNA, and increases STAT3 protein expression in induced Treg (iTreg) cells. (A) Heatmap showing selected differentially expressed genes in iTreg cells under hypoxia and normoxia conditions (H‐N). The average log fold change (logFC) is shown for genes that are statistically significant in RNA‐seq. (B) mRNA expression and chromatin accessibility of *Stat3*. The assay for transposase‐accessible chromatin using sequencing (ATAC‐seq) and RNA‐seq Integrative Genomics Viewer tracks are shown from T helper 17 (Th17) and iTreg cells differentiated under normoxia (N) and hypoxia (H). (C) mRNA expression and chromatin accessibility at the last intron of *Stat3*. The ATAC‐seq and RNA‐seq Integrative Genomics Viewer tracks are shown from Th17 and iTreg cells differentiated under normoxia (N) and hypoxia (H). (D) Representative western blot image of STAT3 (left) and corresponding graph showing relative protein quantification (right) in iTreg cells cultured under normoxia (N) and hypoxia (H) conditions (*n* = 4). β‐actin is used as a protein loading control and sample quantification is normalized to iTreg normoxia. The protein quantification of STAT3 relative to β‐actin (STAT3 : Actin) is indicated under each lane of the representative western blot. The statistical analysis was performed using one‐sample t‐test where *P ≤ 0.05. (E) Representative dot plots (left) and quantification (right) of STAT3 and pSTAT3 from flow cytometry analysis of iTreg cells differentiated under normoxia or hypoxia conditions (*n* = 2, with three technical replicates). After 3 days differentiation, iTreg cells were stimulated without (No IL‐6) and with 60 ng·mL^−1^ of IL‐6 (+IL‐6) for 30 min. Statistical analysis was performed using a paired *t*‐test, where *P ≤ 0.05. (F) Relative *Stat3* mRNA expression detected by qPCR from iTreg cells differentiated for 3 days in normoxia (N) and hypoxia (H) with and without the hypoxia‐inducible factor 1‐alpha (HIF‐1α) inhibitor (HIF‐1αi) YC‐1 (2.5 μm *n* = 2, and 5 μm *n* = 3). mRNA expression is normalized to the corresponding normoxia sample. *Hprt* was used as endogenous control. Data presented as mean ± s.e.m. (G) mRNA expression and chromatin accessibility of *Vdr*. The ATAC‐seq and RNA‐seq Integrative Genomics Viewer tracks are shown from Th17 and iTreg cells differentiated under normoxia (N) and hypoxia (H). (H) Relative *Vdr* mRNA expression detected by qPCR in iTreg cells. mRNA expression in hypoxia (H) is normalized to the corresponding normoxia (N) sample. *Hprt* was used as endogenous control. Data presented as mean ± s.e.m. (*n* = 3). Statistical analysis was performed using a one‐sample *t*‐test where *P ≤ 0.05. (I) mRNA expression and chromatin accessibility of *Ahr*. The ATAC‐seq and RNA‐seq Integrative Genomics Viewer tracks are shown from Th17 and iTreg cells differentiated under normoxia (N) and hypoxia (H). (J) Relative *Ahr* mRNA expression detected by qPCR in iTreg cells. mRNA expression in hypoxia (H) is normalized to the corresponding normoxia (N) sample. *Hprt* was used as endogenous control. Data presented as mean ± s.e.m. (*n* = 3). Statistical analysis was performed using a one‐sample *t*‐test where ‘ns’ is *P* > 0.05. Treg, T regulatory.

Next, we focused on gene expression changes caused by hypoxia and performed pathway enrichment analysis on our RNA‐seq data. Importantly, we found that the expressions of several factors critical for Th17 differentiation were significantly upregulated, including *Stat3*, *Ahr* and *Vdr* (Fig. 6A). STAT3 is a TF that plays an essential role in regulating Th17 cell differentiation [32, 40, 41, 42]. A closer examination of the *Stat3* locus revealed that hypoxia upregulated its expression in iTreg cells, whereas the expression level in Th17 cells remained unchanged (Fig. 6B). ATAC‐seq can provide valuable information on potential regulatory elements located in intronic regions by allowing the detection of differentially accessible peaks at these sites upon exposure to specific stimuli [43], such as hypoxia. Introns can comprise regulatory elements with the potential to alter gene processing and expression [44, 45]. For instance, intron 22 in the *Stat3* gene contains a UG‐rich region that is involved in *Stat3* alternative splicing [46]. Interestingly, our ATAC‐seq data revealed that hypoxia led to a significantly increased peak in the last intron of *Stat3*, intron 23, in iTreg cells. A similar peak was also clearly detected in Th17 cells under both normoxia and hypoxia conditions but was reduced in iTreg cells in normoxia (Fig. 6C). Next, we verified the upregulation of STAT3 expression in iTreg cells at the protein level (Fig. 6D,E) and further confirmed that upon exposure to the inflammatory cytokine IL‐6, iTreg cells differentiated under hypoxia conditions had increased STAT3 phosphorylation, compared with iTreg cells differentiated in normoxia (Fig. 6E). Given that HIF‐1 is the key TF mediating the hypoxic response via stabilization of its oxygen‐sensitive HIF‐1α subunit, we investigated whether the hypoxia‐induced *Stat3* upregulation at the mRNA level was dependent on HIF‐1α. To address this question, we differentiated iTreg cells in the presence of YC‐1, a widely used inhibitor of HIF‐1α that mimics the effects of HIF‐1α silencing and has been used in other fields of research to study HIF‐1α‐dependent mechanisms, [47, 48, 49]. HIF‐1α inhibition did not cause a significant change in *Stat3* mRNA expression (Fig. 6F), suggesting that the upregulation of *Stat3* in hypoxia may not be directly dependent on HIF‐1α. Altogether, this suggests that while HIF‐1α is not directly required to induce *Stat3* expression, additional mechanisms under hypoxia conditions may be at play. Namely, here we observed increased chromatin accessibility in the last intron, which may serve as a regulatory element to facilitate TF binding, thereby contributing to the upregulation of Stat3 expression.

Additionally, our RNA‐seq data also revealed significant upregulation of *Ahr* and *Vdr* by hypoxia in iTreg cells (Fig. 6A,G,I). AHR and VDR are another two TFs with critical functions in Th17 cell differentiation [50, 51, 52, 53]. Then, we performed qPCR analysis of these two genes from different *in vitro* polarized iTreg cells and confirmed the upregulation of these genes by hypoxia in iTreg cells (Fig. 6H,J). Taken together, hypoxia strongly impacted iTreg cell differentiation by modifying chromatin accessibility and influencing the expression of several key factors involved in Th17/Treg cell differentiation.

### Inhibition of HIF‐1α and STAT3 reduces RORγt protein expression but does not upregulate FOXP3 in iTreg cells

**Fig. 7:**
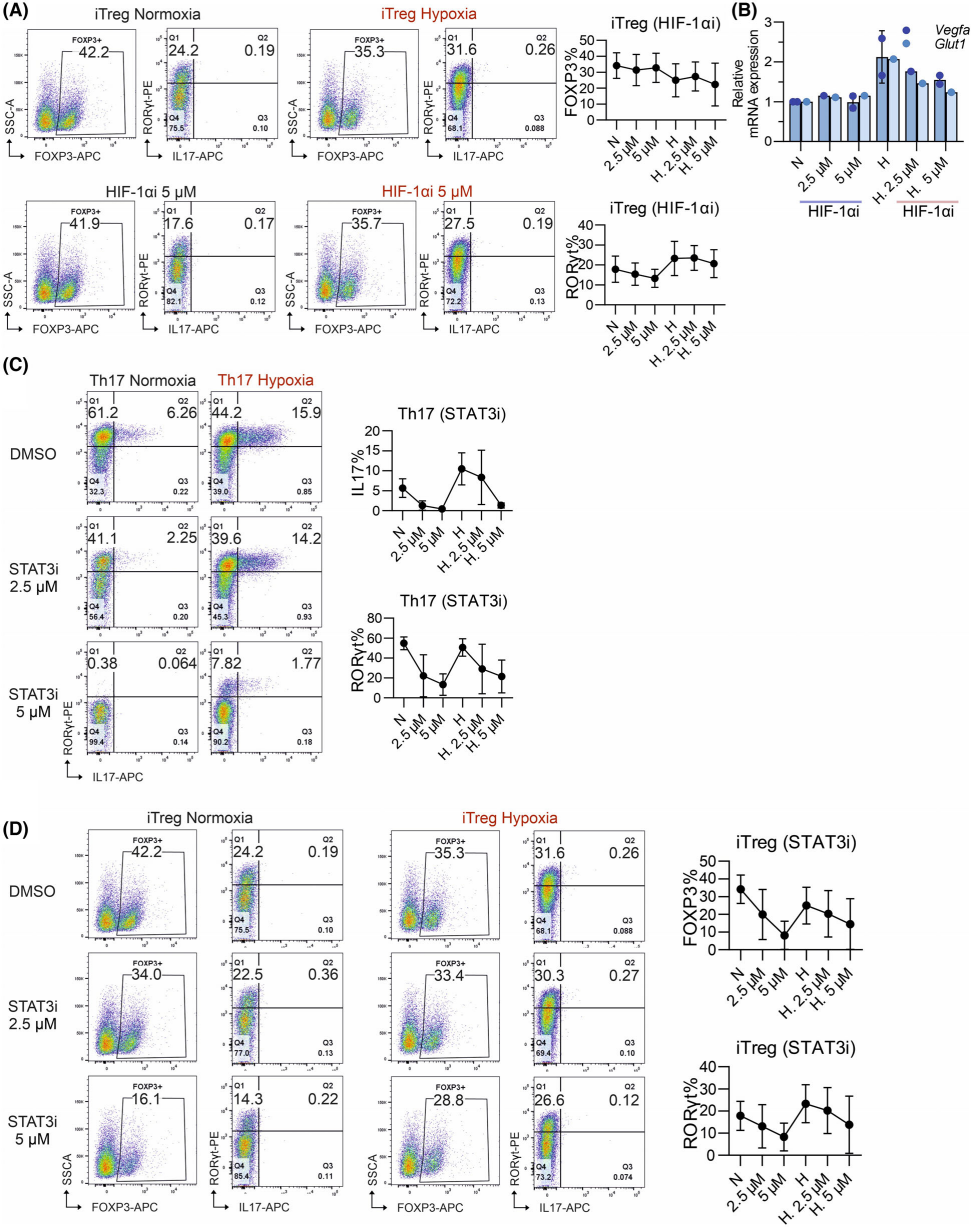
Inhibition of HIF‐1α and STAT3 in hypoxia reduces RORγt expression but does not upregulate forkhead box P3 (FOXP3). (A) Representative dot plots of FOXP3, RORγt, and IL17 (left) and quantification of FOXP3 and RORγt (right) from flow cytometry analysis of induced Treg (iTreg) cells after 3 days differentiation under normoxia (N) or hypoxia (H) with and without the hypoxia‐inducible factor 1‐alpha (HIF‐1α) inhibitor (HIF‐1αi) YC‐1 (2.5 μm *n* = 2, and 5 μm *n* = 3). Data presented as mean ± s.e.m. (B) Relative mRNA expression of *Vegfa* (*n* = 2) and *Glut1* (*n* = 1) detected by qPCR in iTreg cells differentiated for 3 days in normoxia or hypoxia (H) with and without the HIF‐1α inhibitor (HIF‐1αi). mRNA expression is normalized to the corresponding normoxia (N) sample. *Hprt* was used as endogenous control. Data presented as mean ± s.e.m. (C) Representative dot plots (left) and quantification (right) of IL‐17 and RORγt from flow cytometry analysis of T helper 17 (Th17) cells differentiated for 3 days under normoxia or hypoxia (H) conditions with and without the STAT3 inhibitor (STAT3i) Stattic (2.5 μm and 5 μm). Data presented as mean ± s.e.m. (*n* = 3). (D) Representative dot plots of FOXP3, RORγt, and IL17 (left) and quantification of FOXP3 and RORγt (right) from flow cytometry analysis of iTreg cells differentiated for 3 days under normoxia or hypoxia (H) conditions with and without the STAT3 inhibitor (STAT3i) Stattic (2.5 μm *n* = 2 and 5 μm *n* = 3). Data presented as mean ± s.e.m. Treg, T regulatory.

Given that HIF‐1α inhibition did not directly affect hypoxia‐induced *Stat3* mRNA expression in iTreg cells, we investigated whether it would affect FOXP3 expression. Meanwhile, to answer whether HIF‐1α inhibition could push the iTreg differentiation away from a Th17‐like phenotype, we also analyzed RORγt and IL‐17 expressions. iTreg cells differentiated in hypoxia had slightly elevated RORγt expression, although not statistically significant. RORγt expression was not sufficient to induce IL‐17 production, likely related to the low accessibility of the *Il17a* locus in hypoxia iTreg cells, compared with Th17 cells (Fig. 2C). Additional stimulation such as the presence of inflammatory cytokines might be required to further increase IL‐17 locus accessibility and induce IL‐17 production. We observed that in iTreg cells, HIF‐1α inhibition had no effect on FOXP3 and IL‐17 expression. However, the expression of RORγt, the lineage‐specific marker supporting Th17 differentiation [54] was reduced (Fig. 7A), suggesting a partial shift away from the Th17 differentiation program. Although the inhibition of HIF‐1α effectively reduced its transcriptional activity, as indicated by decreased expression of its target genes vascular endothelial growth factor (*Vegf*) and glucose transporter Type 1 (*Glut1*) (Fig. 7B), this was not sufficient to restore FOXP3 expression or fully rescue iTreg differentiation.

To investigate whether elevated STAT3 levels contribute to the suppression of iTreg differentiation under hypoxia, we differentiated Th17 and iTreg cells in the presence of the STAT3 inhibitor Stattic (STAT3i). STAT3 inhibition effectively suppressed Th17 differentiation, as evidenced by a dose‐dependent reduction in RORγt and IL‐17 expression (Fig. 7C). In iTreg cells, STAT3 inhibition also resulted in reduced RORγt levels (Fig. 7D); however, FOXP3 expression remained unchanged under hypoxia, indicating that STAT3 blockade alone was insufficient to restore iTreg differentiation.

### Transcription factor motif analysis uncovers potential transcription factors regulating iTreg cell differentiation in hypoxia

Previous studies have shown the involvement of TFs in chromatin reorganization at key Th subset loci during Th cell activation and differentiation. Therefore, we sought to identify potential TFs that could bind to the open chromatin and may contribute to suppressing iTreg differentiation under hypoxia. The TF motif enrichment was performed by comparing the frequency of TF‐binding sites in the ATAC‐seq peak sequences against the background sequences, and the top enriched motifs were identified.

**Fig. 8:**
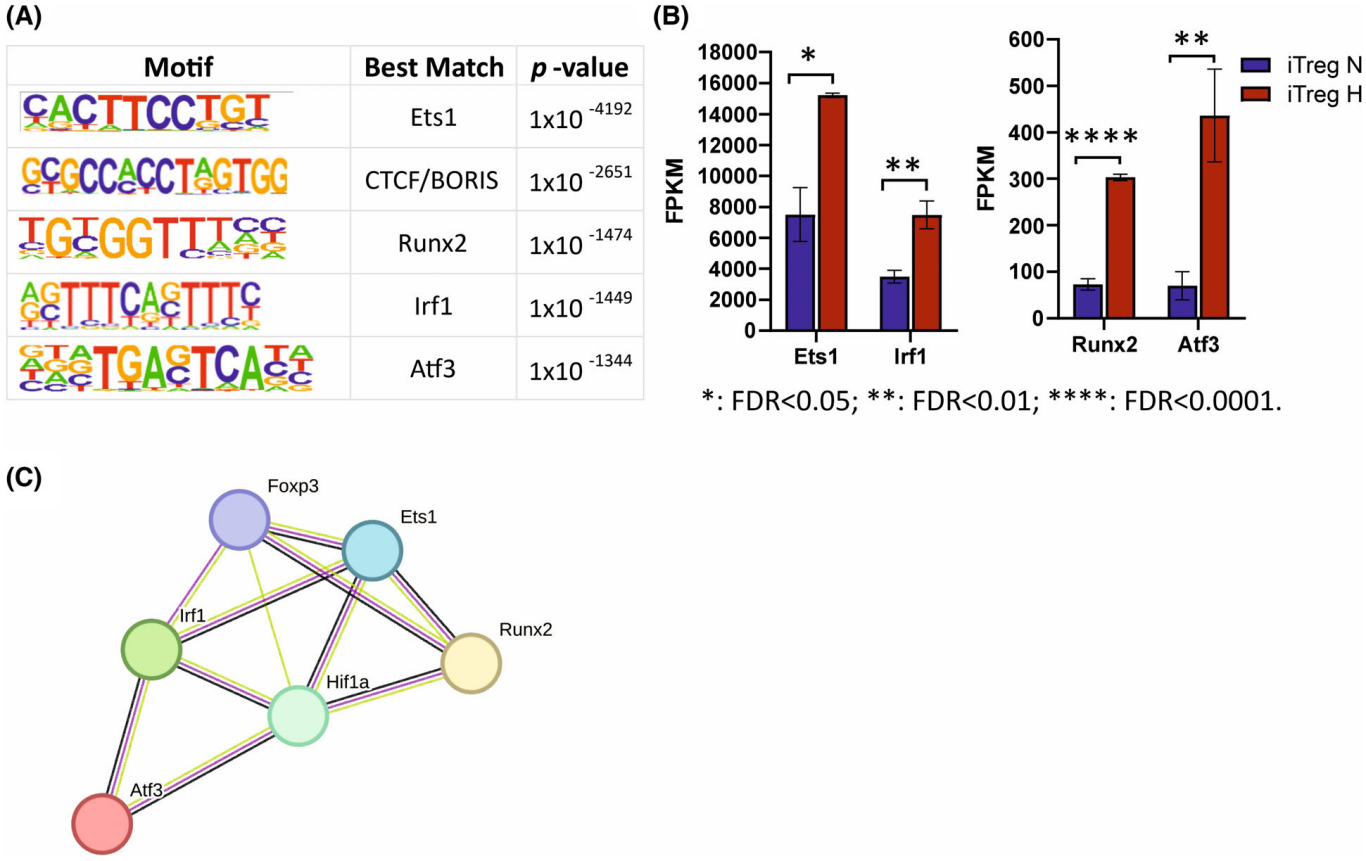
Transcription factor motif enrichment analysis identifies potential transcription factors regulating induced Treg (iTreg) cell differentiation by hypoxia. (A) Hypoxia enriched motifs from assay for transposase‐accessible chromatin using sequencing (ATAC‐seq) motif enrichment analysis of iTreg cells. The Homer software is used to identify the conserved sequence features of the peak enrichment position. HOMER *de novo* motif results are presented. (B) mRNa expression of *Ets1, Irf1, Runx2* and *Atf3* shown as fragments per kilobase million (FPKM) from RNA‐seq analysis of iTreg cells. iTreg hypoxia (H) shown in orange is compared with normoxia (N) shown in blue. Data are shown as mean ± s.e.m. and *: FDR < 0.05; **: FDR < 0.01; ****: FDR < 0.0001. (C) String interaction network analysis of hypoxia enriched motifs with hypoxia‐inducible factor 1‐alpha (HIF‐1α) and forkhead box P3 (FOXP3). Predicted protein–protein interaction network, where experimentally determined interactions are shown by a connecting magenta line, green: text mining (Co‐Mentioned in PubMed Abstracts), blue: co‐expression, magenta: experimentally determined. Treg, T regulatory.

Several TF motifs were enriched under hypoxia of which ETS1, CTCF/BORIS, and IRF1 were among the top enriched motifs in at least two of our three replicates (Fig. 8A). RUNX2 and ATF3 motifs were enriched in one replicate. Importantly, the gene expression of the selected TFs increased in response to hypoxia (Fig. 8B). We then performed STRING interaction analysis and confirmed a possible interaction between the TFs induced by hypoxia in the iTreg cells and HIF‐1α (Fig. 8C). Taken together, these results suggest that the identified TFs may contribute to the regulation of Th17/Treg balance under hypoxia, shifting the differentiation from iTreg cells toward a Th17 phenotype.

## Discussion

Treg and Th17 cells play crucial functions in the immune response. However, disruption of the Treg and Th17 differentiation balance can result in uncontrolled inflammation and immune‐mediated diseases. There are different layers of regulatory mechanisms that ensure appropriate cell differentiation and response. Here, we focused on understanding how hypoxia induces changes in chromatin accessibility and gene expression for modulating the Th17/Treg balance.

Cytokines, such as IL‐6 and IL‐2 induce chromatin remodeling and other epigenetic changes that are required for the Th cell differentiation process [15, 17, 55, 56], but there are additional factors that may impact differentiation. Consistent with previous reports, in our study hypoxia suppressed iTreg and promoted Th17 differentiation [20, 38]. Additionally, our study further demonstrates that hypoxia induces more pronounced transcriptional changes in iTreg cells compared with Th17 cells. Notably, we observed upregulation of *Hif1a* mRNA and a stronger suppression of mitochondrial metabolism in iTreg cells, compared with Th17, highlighting their increased sensitivity to low‐oxygen stress. Furthermore, the effects of hypoxia on FOXP3 expression may be reversible, as the Foxp3 locus appears to remain accessible under both normoxic and hypoxic conditions. This suggests that the transcriptional machinery could potentially re‐engage once normoxia is restored. However, further experimental validation is necessary to determine the extent to which the iTreg phenotype can be re‐established following hypoxic exposure, particularly in terms of FOXP3 stability, suppressive function, and epigenetic integrity.

When oxygen availability in the microenvironment decreases, cells activate survival mechanisms largely driven by HIF signaling, such as changes in metabolism [57]. Metabolism plays a critical role in driving forward Th cell processes, including Th cell activation, differentiation, and function [58, 59]. Consequently, manipulating cellular metabolism has emerged as a potential therapeutic strategy to fine‐tune T‐cell responses in inflammatory disease. A study reported that early, short‐term inhibition of mitochondrial metabolism with oligomycin can direct Th cell differentiation toward iTreg instead of Th17 [60]. Our study showed that prolonged exposure to hypoxia results in reduced OXPHOS both in Th17 and iTreg cells but does not enhance FOXP3 expression. In line with our results, other studies have reported that OXPHOS supports Treg suppressive function [61, 62] and that increased glycolytic metabolism negatively regulates FOXP3 expression and stability [63, 64, 65]. Future research would benefit from further defining whether different functional properties can be achieved by manipulating iTreg metabolism either at the early stages of activation and differentiation, or during long‐term differentiation cultures and expansion. The timepoint and strategy by which metabolism is manipulated could have important consequences that will define the potential for future clinical applications.

Given that hypoxia also alters chromatin accessibility in cancer cells [22], we asked whether some of the changes induced under hypoxia could bias Th cell differentiation toward a Th17 phenotype. Using ATAC‐seq we looked at changes in the chromatin accessibility landscape upon Th17 and iTreg differentiation under hypoxia. As an important feature of the iTreg response to hypoxia, compared with Th17, *Hif1a* was significantly upregulated already at the transcriptional level. Our ATAC‐seq analysis showed that the *Hif1a* locus accessibility is comparable in normoxia and hypoxia. This suggests that hypoxia does not regulate *Hif1a* expression by altering chromatin accessibility. Motif enrichment analysis allowed the identification of TF‐binding motifs that were enriched under hypoxia and in the Th17 condition, suggesting that they could be involved in modulating the iTreg and Th17 balance. Enrichment of the CTCF/BORIS‐binding motif was induced by hypoxia in iTreg cells. CTCF and BORIS are TFs involved in the formation of chromatin structures to regulate gene transcription [66]. Involvement of these two factors suggests increased chromatin remodeling in hypoxia. In line with this finding, IRF1 and ETS1, TFs involved in chromatin remodeling during Th cell differentiation [67, 68, 69, 70, 71], were upregulated by hypoxia in iTreg. This suggests that they may participate in chromatin remodeling events that modulate iTreg differentiation by hypoxia.

Among the changes induced by hypoxia in iTreg cells, we observed that both *Stat3* and *Hif1a* were significantly upregulated. These TFs are involved in Th differentiation. *Hif1a* mRNA is upregulated during Th17 differentiation under normoxia conditions [20], which is further enhanced by hypoxia [38, 72, 73]. Interestingly, crosstalk between Stat3 and Hif1a has been previously reported in cancer and inflammation [74, 75, 76]. In CD4+ T cells, STAT3 activation increases *Hif1a* at the RNA level [20, 73]. STAT3 can physically interact with HIF‐1α, and cooperate in the regulation of HIF target genes [74], and is involved in the regulation of HIF‐1α by inhibiting protein degradation [77]. Importantly, STAT3 and HIF‐1α crosstalk is implicated in inflammatory diseases [78]. While interaction between STAT3 and HIF‐1α signaling has been reported in the literature, in our study, HIF‐1α inhibition did not lead to *Stat3* mRNA level changes under hypoxia, suggesting that *Stat3* induction in hypoxia may not solely be dependent on HIF‐1α. Importantly, our data revealed that under hypoxia, in iTreg cells, the increased STAT3 protein expression enabled enhanced STAT3 phosphorylation in response to exposure to the inflammatory cytokine IL‐6, which may have significant implications for iTreg function in inflammatory settings *in vivo*.

Hypoxia also induced changes in *Stat3* chromatin accessibility. STAT3 has two main isoforms, STAT3α and STAT3β, that arise from alternative splicing at exon 23 [79, 80]. The STAT3β isoform can act as a dominant‐negative regulator of STAT3α‐mediated transcriptional activity. To the best of our knowledge, while the acceptor site required for STAT3β alternative splicing has been mapped to exon 23 [81], additional regulatory elements controlling this splicing event remain poorly defined. A recent study [46] identified UG‐rich elements within intron 22 that facilitate binding of CUG‐binding protein 2 (CUGBP2), promoting splicing in favor of the STAT3β isoform. Based on our knowledge, our data provided the first evidence that hypoxia increased chromatin accessibility at intron 23 of the *Stat3* locus, suggesting a potential novel regulatory mechanism influencing STAT3 splicing under hypoxia conditions. This finding indicates that hypoxia may influence alternative splicing not only through transcriptional regulation but also by modulating the local chromatin landscape. Given the demonstrated elevated STAT3 protein levels and enhanced IL‐6–induced phosphorylation observed in hypoxic iTreg cells, the shift in accessibility of Stat3 could have functional consequences for the balance between STAT3 isoforms. This may potentially affect downstream signaling and transcriptional programs. These findings open new avenues for exploring how‐oxygen availability shapes immune cell fate through epigenetic and post‐transcriptional mechanisms.

In conclusion, this study explores how hypoxia reshapes chromatin accessibility and contributes to transcriptional changes that influence the Th17/Treg balance. Our integrated ATAC‐seq and RNA‐seq analyses resulted in the identification of TFs that are upregulated by hypoxia and may regulate iTreg differentiation. Importantly, we observed that hypoxia increased chromatin accessibility at the *Stat3* locus in iTreg cells, with possible consequences for iTreg cell differentiation. Previous studies have reported that HIF‐1α influences Th17/Treg differentiation by directly regulating IL17/RORγt expression and Foxp3 stability [20]. Here, we report a novel mechanism that the crosstalk between STAT and hypoxia signaling modulates Th17/iTreg differentiation. Altogether, our study provides new insight into the mechanisms that regulate Th17/Treg balance under hypoxia.

## Materials and methods

### Naïve T‐cell isolation and *in vitro* Th differentiation

C57BL/6 mice (The Laboratory Animal Center, University of Oulu) were maintained in the Oulu Laboratory Animal Centre at the University of Oulu following Finnish legislation and in accordance with European Directive 2010/63/EU on the protection of animals used for scientific purposes. Mice were housed in open top cages (two to five mice per cage) supplied with nesting material and cardboard tunnels as enrichment. Chow diet and tap water were provided *ad libitum*. The study and all experimental procedures were performed in accordance with the license number ESAVI/7374/2019 and ESAVI/30556/2024, approved by the National Project Authorization Board of Finland. Naive CD4+ T cells were isolated from the spleen and lymph nodes of 3–6‐month‐old female C57BL/6 mice using a CD4+ T Cell Isolation Kit (130‐104‐454; Miltenyi Biotec; Bergisch Gladbach, Germany), followed by positive selection with CD62L (L‐selectin) MicroBeads (130‐049‐701; Miltenyi Biotec).

For iTreg cell differentiation, naïve T cells were activated with 1 μg·mL^−1^ of plate‐bound anti‐CD3 (16‐0031‐86; eBioscience; San Diego, CA, USA), and anti‐CD28 (16‐0281‐85; eBioscience), and cultured for 3 days in RPMI 1640 Medium (RPMI, 31870‐025; Gibco, Thermo Fisher Scientific; Waltham, MA, USA) supplemented with 10% fetal bovine serum (FBS), 100 IU·mL^−1^ penicillin (P0781‐100ML; Sigma‐Aldrich), 0.1 mg·mL^−1^ streptomycin (P0781‐100ML; Sigma‐Aldrich), 2 mm glutamine (G7513‐100ML; Sigma‐Aldrich; St. Louis, MO, U), and 2.5 μm β‐Mercaptoethanol (31350‐010; Gibco). The cells were cultured with 10 ng·mL^−1^ IL‐2 (202‐IL; R&D Systems; Minneapolis, MN, USA) and 10 ng·mL^−1^ TGF‐β1 (100‐21; Peprotech; Cranbury, NJ, USA).

For Th17 differentiation, naïve T cells were activated with 5 μg·mL^−1^ of plate‐bound anti‐CD3 and 0.5 μg·mL^−1^ of plate‐bound anti‐CD28. The cells were cultured for 3 days in Iscove's modified Dulbecco's medium (IMDM; 21980‐032; Gibco, Thermo Fisher Scientific) supplemented with 10% FBS, 100 IU·mL^−1^ penicillin, 0.1 mg·mL^−1^ streptomycin, 2 mm glutamine, 2.5 μm β‐Mercaptoethanol, 60 ng·mL^−1^ IL‐6 (216‐16; Peprotech), 5 ng·mL TGF‐β1 (100‐21; Peprotech), 1 μg·mL^−1^ anti‐IFNγ (500‐P119; Peprotech), and 30 ng·mL^−1^ IL‐23 (200‐23; Peprotech).

For the hypoxia condition, the cells were differentiated in a BBD 6220, Heraeus incubator (Thermo Scientific) with 5% oxygen. For the normoxia condition, the cells were differentiated with 21% oxygen.

For inhibitor experiments, the HIF‐1α inhibitor YC‐1 (cat. no.: ALX‐420‐025 M001; Enzo Life Sciences; Farmingdale, NY, USA) or STAT3 inhibitor Stattic (cat. no.: T6308; TargetMol; Boston, MA, USA) was added (2.5 or 5 μm) at the beginning of the differentiation cultures and every time media was added. The control condition containing DMSO was included and used for both STAT3 and HIF‐1α inhibitor experiments.

### Flow cytometry

For analysis of FOXP3, RORγ, IFNγ, and IL‐17, cells were stimulated for 4 h at 37 °C with Phorbol 12‐Myristate 13‐Acetate, Ionomycin, and Brefeldin A solution followed by fixation and permeabilization with the Foxp3/Transcription Factor Staining Buffer Set (cat. no.: 00‐5523‐00; eBioscience). The cells were stained with antibodies against FOXP3‐PE (cat. no.: 12‐4774‐42, clone 150D/E4; eBioscience), FOXP3‐AlexaFluor647 (cat no.: 560401, clone MF23; BD Bioscience, Milpitas, CA, USA), ROR gamma (t)‐PE (cat. no.: 12‐6988‐82, clone AFKJS‐9; eBioscience), IL‐17A‐APC (cat. no.: 17‐7177‐81, clone eBio17B7; eBioscience), IL‐17A‐PE (cat. no.: 559502, clone TC11‐18H10; BD Bioscience), IFNγ‐AlexaFluor488 (cat. no.: 557724, clone XMG1.2; BD Bioscience), and were analyzed with the LSRFortessa™ flow cytometer (BD Biosciences).

For STAT3 and pSTAT3 analysis, cells were incubated at 37 °C for 30 min with and without IL‐6 (60 ng·mL^−1^, 216‐16; Peprotech). The cells were fixed with 4% formaldehyde (cat. no.: 28908; Pierce™ 16% Formaldehyde) and permeabilized with 100% methanol prior to staining with BD Phosflow STAT3 (pY705)‐AlexaFluor647 (cat. no.: 557815, clone 4/P‐STAT3; BD Bioscience) and STAT3‐PE/Cyanine7 (cat. no.: 678010, clone 4G4B45; BioLegend; San Diego, CA, USA).

### Immunoblotting

Th0, Th17, and iTreg cells were collected after differentiation for 3 days in normoxia and hypoxia. Samples were lysed with detergent buffer containing complete protease inhibitors. Samples were electrophoresed, transferred to PVDF membranes (Bio‐Rad; Hercules, CA, USA), and immunoblotted with antibodies for HIF‐1α (36169; Cell Signaling Technology; Danvers, MA, USA), STAT3 (9139; Cell Signaling Technology), and β‐Actin (A5441; Sigma‐Aldrich). Image analysis for relative quantification was performed with FiJi (imagej) version 10.2023, and statistical analysis was performed with graphpad prism 10.

### RNA‐seq library preparation, sequencing, and analysis

*In vitro* differentiated Th0, Th17, and iTreg cells in normoxia and hypoxia were collected and processed for RNA extraction with the RNeasy Mini Kit (cat. no.: 74104; Qiagen; Venlo, The Netherlands) according to the manufacturer's protocol, and 600 ng of RNA was prepared for sequencing. Sequencing libraries were generated using NEBNext® Ultra RNA Library Prep Kit for Illumina® (NEB, USA) according to the manufacturer's instruction. RNA‐seq with paired‐end 150‐nt read length was performed at Novogene (Cambridge, UK) with the Illumina NovaSeq 6000 instrument. About 20 million paired‐end reads per sample were obtained. Clean data were obtained by removing reads containing adapter and poly‐N sequences and reads with low quality from raw data. All the downstream analyses were based on the clean data.

### ATAC‐seq library preparation, sequencing, and analysis

A total of 100 000 cells cultured under Th0‐, Th17‐, and iTreg‐polarizing conditions in normoxia and hypoxia were collected, and libraries were prepared with the ATAC‐seq Kit (Active Motif, Carlsbad, CA) following the manufacturer's instructions until tagmentation and purification; then, a 5‐min extension in PCR at 72 °C was performed. NGS and data analysis were performed at Novogene (UK). After trimming adaptor sequences and filtering low‐quality sequences, BWA was used for the genome mapping analysis for the clean reads [82]. Peak calling was performed using the macs2 software [83], threshold *q* value of 0.05 was used to call peaks. Peak annotation was performed with PeakAnnotator [84]. The homer software was used to identify the conserved sequence features of the peak enrichment position.

### Quantitative RT‐PCR

RNA was extracted from *in vitro* cultured Th0, Th17, and iTreg cells using the RNeasy Mini Kit (74104; Qiagen), and the cDNA synthesis was performed using the Superscript cDNA synthesis kit (11754‐050; Invitrogen; Carlsbad, CA, USA). Quantitative PCR was performed using the LUNA universal qPCR Master Mix (M3003L; New England Biolabs; Ipswich, MA, USA). The corresponding primer sequences are in Table 1. The *Hprt* gene was used as an endogenous control.

**Table 1:** Primer sequences for quantitative RT‐PCR.

Gene	Forward primer 5′‐3′	Reverse primer 5′‐3′
*Hprt*	GGCCAGACTTTGTTGGATTTG	CGCTCATCTTAGGCTTTGTATTTG
*Vdr*	GAATGTGCCTCGGATCTGTGG	ATGCGGCAATCTCCATTGAAG
*Ahr*	AGCCGGTGCAGAAAACAGTAA	AGGCGGTCTAACTCTGTGTTC
*Stat3*	GTCGGTTGGAGGTGTGAGGTAG	AGGGTCTGGAGTCTGGGTTGG
*Vegfa*	GCACTGGACCCTGGCTTTAC	AACTTGATCACTTCATGGGACTTCT
*Glut1*	TCAAACATGGAACCACCGCTA	AAGAGGCCGACAGAGAAGGAA

### Seahorse XFe96 extracellular flux analysis

All Seahorse XFe96 assays were performed following the manufacturer's protocol. Briefly, after cell differentiation for 3 days, as described above, 250 000–300 000 cells per well were plated on a Poly‐*d*‐Lysine (MP Biomedicals; Irvine, CA, USA) coated 96‐well plate. For the assay, XF RPMI assay media, pH 7.4 (cat. no.: 103576‐100; Agilent Technologies) was supplemented with 10 mm Seahorse XF Glucose (cat. no.: 103577‐100; Agilent Technologies), 1 mm Seahorse XF Pyruvate (cat. no.: 103578‐100; Agilent Technologies) and 2 mm Seahorse XF Glutamine (cat. no.: 103579‐100; Agilent Technologies). The Mito Stress Assay was performed with four sequential injections of Oligomycin (1.5 μm, cat. no.: O4876‐5MG; Sigma‐Aldrich), FCCP (1 μm, cat. no.: ab120081; Abcam; Cambridge, UK), FCCP (0.5 μm), Rotenone (0.5 μm, cat. no.: R8875‐1G; Sigma‐Aldrich) + Antimycin A (0.5 μm, cat. no.: A8674‐25MG; Sigma‐Aldrich). The Glycolytic Rate Assay was performed with two sequential injections of Rotenone (0.5 μm) + Antimycin A (0.5 μm), and 2‐Deoxy‐*d*‐glucose (50 mm, 2‐DG, cat. no.: D6134‐5G; Sigma‐Aldrich). Data were normalized according to cell numbers for equal comparison among conditions. Mito Stress assay analysis was performed according to Agilent's guidelines using the highest signal from FCCP injection, and Glycolytic Rate Assay analysis was performed with the Seahorse Analytics cloud analysis portal.

## Conflicts of interest

The authors declare no conflict of interest.

## Author contributions

MC‐O performed most of the experiments, interpreted the results, and prepared the manuscript. SS performed the bioinformatics analysis. SY and TQ performed the experiments. BL took part in the analysis and supervised the study. ZC performed the analysis. ZC conceptualized and supervised the study, took part in the analysis, interpretation and visualization of results, and reviewed the manuscript.

## Supporting information


**Table S1.** Differentially expressed genes (DEG) in Th17 cells under hypoxia compared to normoxia conditions from RNA‐seq analysis.
**Table S2.** Differentially expressed genes (DEG) in iTreg cells under hypoxia compared to normoxia conditions from RNA‐seq analysis.
**Table S3.** Differential peaks associated with genes in Th17 cells under hypoxic versus normoxic conditions from ATAC‐seq analysis.
**Table S4.** Enriched HIF‐1 pathway genes in Th17 cells under hypoxic versus normoxic conditions from ATAC‐seq analysis.
**Table S5.** KEGG pathway enrichment analysis of Treg upregulated genes in hypoxia including the top 25 enriched pathways.
**Table S6.** KEGG pathway enrichment analysis of Treg downregulated genes in hypoxia including the top 25 enriched pathways.
**Table S7.** Differential peaks associated with genes in iTreg cells under hypoxic versus normoxic conditions from ATAC‐seq analysis.

## Data Availability

The datasets generated and analyzed during the current study are available from the NCBI SRA database with the dataset identifier PRJNA1292796.
